# Grain Refinement Mechanism of 5A06 Aluminum Alloy Sheets during Cold Rotary Forging

**DOI:** 10.3390/ma16072754

**Published:** 2023-03-29

**Authors:** Xinghui Han, Wenyi Chen, Xuan Hu, Lin Hua, Fang Chai

**Affiliations:** 1 School of Materials Science and Engineering, Wuhan University of Technology, Wuhan 430070, China; hanxinghui@whut.edu.cn (X.H.);; 2 Hubei Key Laboratory of Advanced Technology for Automotive Components, Wuhan University of Technology, Wuhan 430070, China

**Keywords:** 5A06 aluminum alloy, cold rotary forging, EBSD, grain refinement mechanism

## Abstract

This paper studies the grain refinement mechanisms of 5A06 aluminum alloy sheets in cold rotary forging (CRF). The results show that the grains are clearly refined from 25.1 µm to 11.8 µm during the CRF process. The grain refinement mechanism can be divided into two modes: (1) The grains with a small Schmid factor (SF) are activated by multi-slip systems, and dense dislocations are segregated along the boundaries of interior regions with different slip systems, which results in a rapidly increasing strain localization along these boundaries. Since the strain localization restrains the coordinate slip deformation between different interior regions, the grains are directly separated into several finer grains. (2) The grains with a large SF are primarily activated by a single slip system, and the dislocation migrates smoothly along most microband boundaries. Then, a more severe lattice rotation causes a transformation to a hard orientation and multi-slip system activation, which contributes to an increase in the rapid misorientation across microband boundaries and thus promotes significant SF grain refinement.

## 1. Introduction

Owing to the urgent demands for lightweight components in the aerospace and automotive fields, sheet components integrating different materials, structures and functions have been widely applied. Usually, these sheet components are manufactured with lightweight and high strength materials, such as aluminum alloy, magnesium alloy and titanium alloy. Meanwhile, these sheet components have complicated geometries, with ribs to improve the stiffness and bosses to install functional units. Typical examples of complicated sheet components are aircraft panels, battery pack shells of new energy vehicles and 3C shells. How to efficiently manufacture these complicated sheet components with a high performance has become a topical and difficult area. Metal forming, which can achieve refined grains and continuous metal flow lines, is an efficient manufacturing process for metal components with high performance [1,2,3,4,5,6,7]. In recent years, much research has been carried out to investigate the grain refinement mechanism of high strength aluminum alloys during severe plastic deformation processes. Oryina et al. [8] studied the grain refinement mechanism of an Al–Zn–Mg alloy during ECAP, finding that a high dislocation density in the elongated grain structure could promote new grain boundary formation in prior grains. It was also found that micro-shear band formation had a strong influence on grain refinement in aluminum alloys during severe plastic deformation. Wu et al. [9] studied the grain refinement mechanism of 7075 aluminum alloy under HPT and found that kinking band generation improved dislocation conversion along slip traces, which contributed to grain boundary formation. Zuo et al. [10] found that dislocation rearrangement and low angle grain boundary transitions had important consequences on the grain refinement of 7075 aluminum alloy during severe plastic deformation. Dislocations and grain boundaries have a significant impact on grain refinement. Moshtaghi et al. [11] studied the effect of dislocations and deformation-induced boundaries on alloys during cold rolling. The study found that GND mainly increased the density of high-angle boundaries by arranging cell block and lamellar boundaries in an 80% cold-rolled specimen. In 50% cold-rolled and 80% cold-rolled specimens, the density of HAGBs increased with an increase in reduction, while the LAGB density did not significantly change. However, it is not easy to manufacture complicated sheet components in traditional overall forming processes, including stamping and forging, owing to the unfeasible metal flow when at low component thicknesses. At present, complicated sheet components are generally manufactured by the milling process, which cannot satisfy the manufacturing requirements of a high performance and a high efficiency.

**Figure 1:**
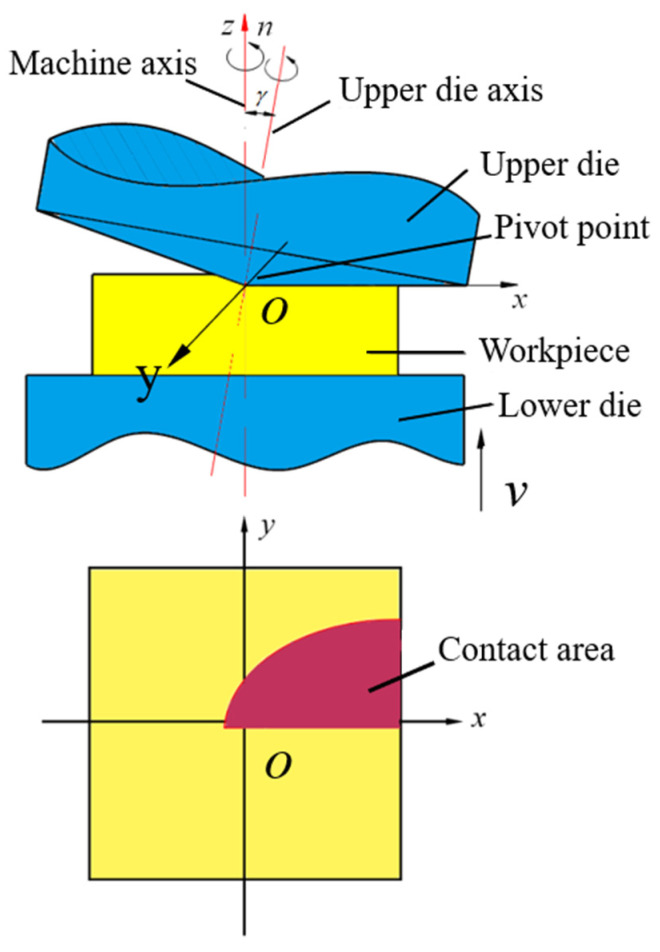
Principle of cold rotary forging.

Cold rotary forging (CRF) is especially suitable for manufacturing complicated sheet components because metal flow is easy in the continuous and local plastic deformation mode. During the CRF process shown in Figure 1, the conical upper die oscillates spatially and the lower die feeds vertically. Under the repeated pressing of two dies, the workpiece produces multi-directional metal flow and forms complicated sheet components at room temperature. By now, some research has been carried out on the CRF process. In view of macroscopic plastic deformation mechanisms, the stress, strain, metal flow and geometry evolution mechanisms during the CRF process of cylinder and ring workpieces have been revealed, and it was found that increasing the deformation speed or decreasing the workpiece thickness could improve the deformation homogeneity [12,13,14,15,16,17]. Metal flow mechanisms during the CRF process of complicated sheet components have been revealed and the process design methods have been developed to achieve efficient manufacturing of typical sheet components, such as tailored sheet components with two-sided bosses [18,19], sheet components with ribs [20], mobile phone shells [21,22] and thin-walled gear [23]. In view of microstructure evolution mechanisms, the CRF process of flange components from Inconel 718 material has been researched, and it was found that the grain size decreased and the hardness value increased [24]. The CRF process of bevel gears from 20CrMnTi material has also been researched, and it was found that refined grains and continuous metal flow lines could be obtained [25]. In summary, the above research indicates that CRF has huge potential in manufacturing sheet components with complicated geometries, but comprehensive research on the microstructure evolution mechanisms during CRF are scarce, which restricts CRF application for manufacturing sheet components with control of geometries and microstructures. Owing to its superior comprehensive properties such as “high specific strength” and “high toughness and plasticity”, 5A06 aluminum alloy is frequently employed as the optimal material for “light-weighting” in the aerospace and automotive industries. Grain refinement has an important impact on alloy properties. Studying the refinement mechanism of different types of grains during CRF can provide an effective theoretical basis for the evolution of subsequent properties. Therefore, this paper systematically studies the grain refinement mechanism of 5A06 aluminum alloy sheets by CRF, which provides an important foundation for further studies on the coordinate control of geometries and microstructures during the CRF process of complicated sheet components.

## 2. Experimental Procedure

**Figure 2:**
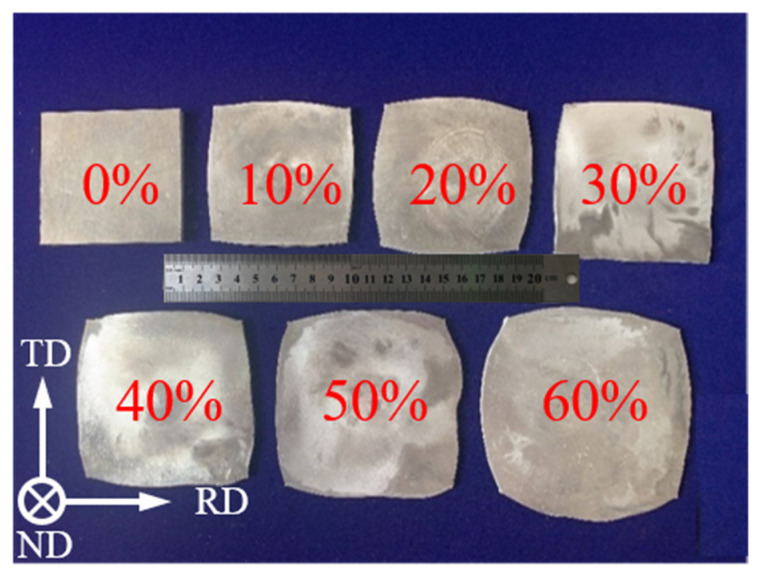
Deformed 5A06 aluminum alloy sheets at different CRF deformation amounts.

The principal aim of the present study is to investigate the grain refinement mechanism of high strength aluminum alloys during the CRF process. The material used in this study is a commercial 5A06 aluminum alloy sheet with the size of 100 × 100 × 10 mm^3^ (length × width × thickness), and the chemical composition is shown in Table 1. The 5A06 aluminum alloy sheets were held in a heated furnace at 345 °C for 2 h for softening annealing. The CRF deformation of the as-annealed 5A06 aluminum alloy sheets was conducted in a T-630 rotary forging machine (Schmid Co. Ltd, Eschlikon, Switzerland). The conical upper die had a swing angle of 2° and rotated with a special motion track with a rotation speed of 25 rad/s. The lower die moved in a straight line along the vertical direction and gradually approached the upper die at a speed of 1 mm/s. The deformation amount was determined by the thinning of the 5A06 aluminum alloy sheet before and after CRF. The CRF deformation amount ranged from 10% to 60%, and 5A06 aluminum alloy sheets with different deformation amounts are shown in Figure 2. Microstructural observations were carried out on aluminum samples with different CRF deformation amounts using a Zeiss Ultra Plus electron microscope (Zeiss Co. Ltd, Oberkochen, Germany) equipped with an EBSD detector. The EBSD observed samples that were prepared by mechanical polishing and electrolytic polishing with a 10% HClO_4_ + 90% C_2_H_5_OH solution. The collected EBSD data were analyzed by HKL channel 5 software version 5.0 beta (Oxford Instruments, Oxford, UK).

## 3. Results and Discussion

**Figure 3:**
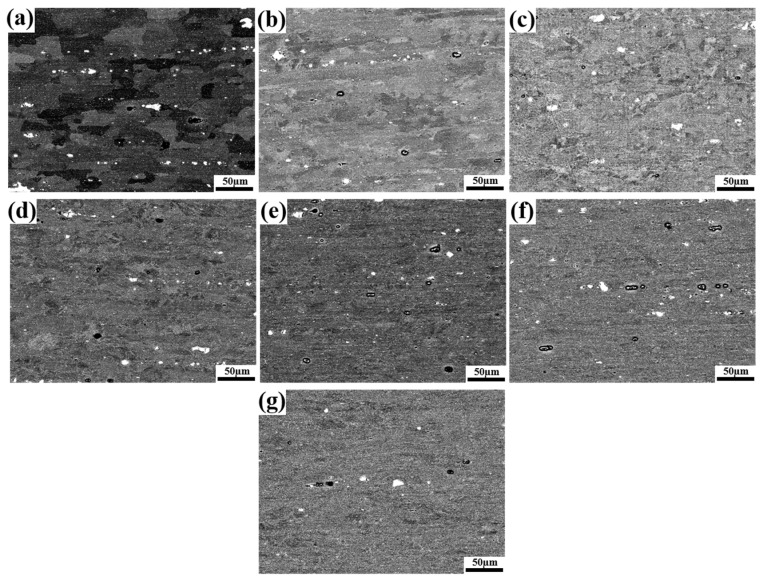
SEM maps of 5A06 aluminum alloy sheets under different CRF deformation amounts: (**a**) as-annealed, (**b**) 10%, (**c**) 20%, (**d**) 30%, (**e**) 40%, (**f**) 50% and (**g**) 60%.

**Figure 4:**
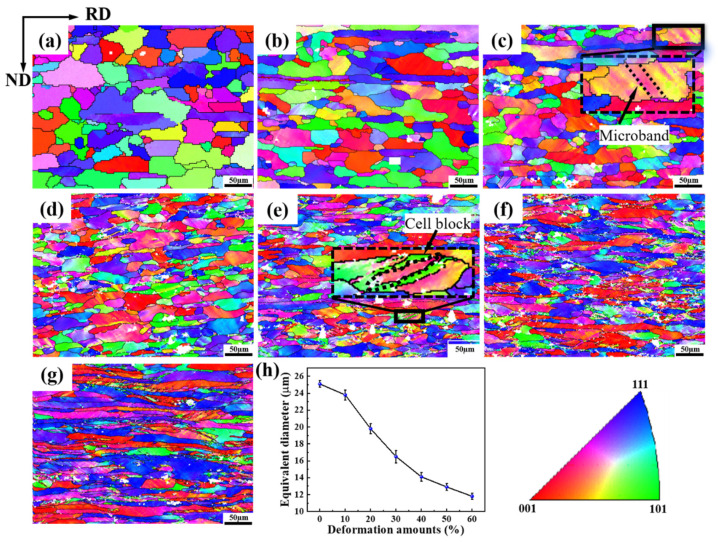
Grain orientation maps under different CRF deformation amounts: (**a**) as-annealed, (**b**) 10%, (**c**) 20%, (**d**) 30%, (**e**) 40%, (**f**) 50%, (**g**) 60% and (**h**) equivalent grain size.

Figure 3a–g shows the SEM maps of 5A06 aluminum alloy sheets under different CRF deformation amounts. It can be clearly seen that the grain morphology changes from block-shaped to long-strip-shaped along the RD. In order to further study the details of grain refinement, EBSD maps were used for analysis. Figure 4a–g shows the grain orientation maps of 5A06 aluminum alloy sheets with different CRF deformation amounts. Figure 4h shows the equivalent grain size. It can be seen that the grain structures exhibit gradual elongation and refinement with an increase in the CRF deformation amount. In the as-annealed condition, the grains with an equivalent size of 25.1 µm are near-equiaxed, and the lattice orientations inside each grain are almost uniform. Moreover, HAGBs are dominant along all grain boundaries, and the overall grain orientations are random. Under CRF-10% and CRF-20% (deformation amount of 10% and 20%, respectively) conditions, parallel microbands form in some matrix grains, which indicates the occurrence of slip behaviors under these deformation conditions. Owing to these slip behaviors, large number of LAGBs are formed. However, the overall grain structures are still near-equiaxed and the grain size slightly decreases. The overall grain orientations are also similar to those under as-annealed conditions. Under CRF-30% and CRF-40% conditions, the grain structures exhibit drastic elongation along the RD, and individual parts of each grain transform toward different orientations, leading to the formation of long cell blocks. Moreover, a large number of both HAGBs and LAGBs form. The equivalent grain size under these two deformation conditions decreases to 16.5 µm and 14.1 µm, respectively. Under CRF-50% and CRF-60% conditions, the slip behavior becomes more severe and the cell blocks become twisted. The equivalent grain size finally decreases to 11.8 µm under CRF-60% conditions. Moreover, compared to the HAGBs, the LAGBs increase more quickly.

**Figure 5:**
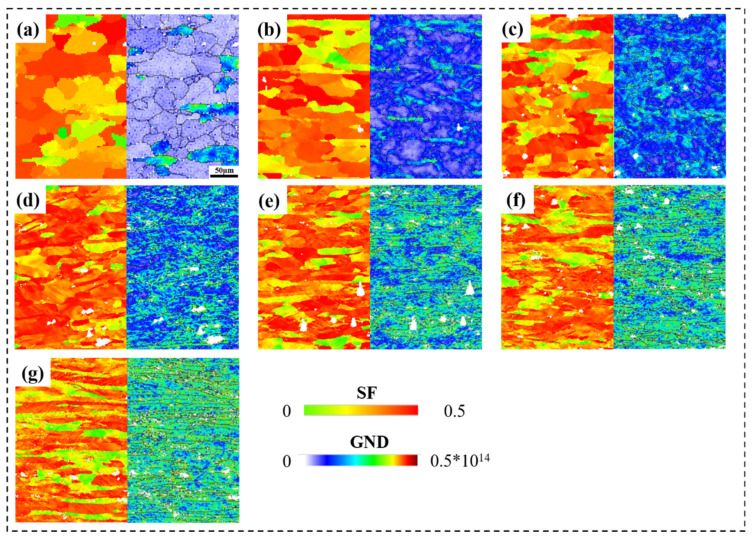
SF and GND maps at different CRF deformation amounts: (**a**) as-annealed, (**b**) 10%, (**c**) 20%, (**d**) 30%, (**e**) 40%, (**f**) 50%, and (**g**) 60%.

**Figure 6:**
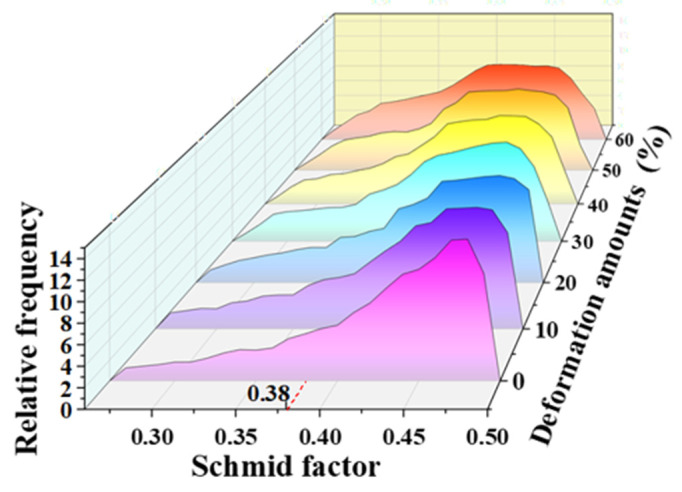
SF value distribution at different CRF deformation amounts.

**Figure 7:**
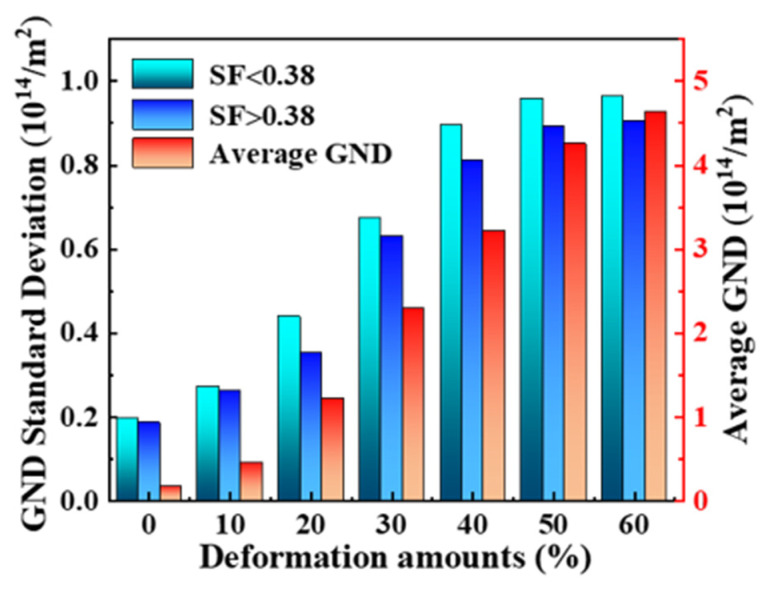
Average GND and GND standard deviation at different CRF deformation amounts.

To investigate the grain refinement mechanism, the evolution of the Schmid factor (SF) and geometrically necessary dislocation (GND) of 5A06 aluminum alloy sheets are analyzed, as shown in Figure 5. It can be seen from Figure 5 that with an increasing CRF deformation amount, the number of large SF grains gradually decrease and the GND primarily increases in some grains and then uniformly increases in most grains. The fraction of grains with different SFs is quantitatively analyzed, as shown in Figure 6. As can be seen, the SF of all grains ranges from 0.26 to 0.5, and grains with SF = 0.45 are the maximum fraction during the entire CRF process. With an increasing deformation amount, the fraction of SF = 0.45 grains and the average SF gradually decrease. According to previous research [26,27], the slip system activation in small SF grains should overcome higher critical resolved shear stresses (CRSS) compared to large SF grains. Therefore, a small SF and a large SF can be regarded as a hard orientation and a soft orientation, respectively. Moreover, difficult slip system activation in small SF grains would cause non-uniform slip deformation in their interior regions. Therefore, the GND standard deviation values of large SF and small SF grains were calculated to quantitatively analyze the deformation non-uniformity during the CRF process, as shown in Figure 7. In this study, based on the range of the SF under all deformation conditions, 0.38 is taken as the critical value, and an SF larger than 0.38 is considered to be a large SF, and vice versa. It can be seen that the GND standard deviation values of both small SF grains and large SF grains primarily increase linearly until the deformation amount reaches 50%, and then become relaltively stable, which indicates that all grains exhibit non-uniform slip deformation before a deformation of 50% and that a uniform severe slip deformation would occur in most grains during the subsequent CRF process. It can be also seen that the GND standard deviation values of small SF grains are constantly higher than those of large SF grains, which indicates that in small SF grains, slip deformation is unfavorable, which results in deformation non-uniformity in these grains.

**Figure 8:**
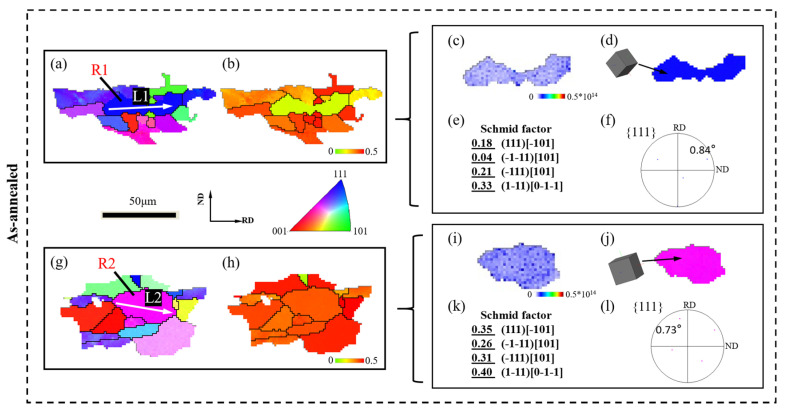
EBSD analysis of local area under as-annealed conditions: (**a**) IPF map of large SF grains, (**b**) SF map of large SF grains, (**c**) GND map of large SF grains, (**d**) grain orientation of large SF grains, (**e**) SF value of large SF grains, and (**f**) PFs of large SF grains; (**g**) IPF map of small SF grains, (**h**) SF map of small SF grains, (**i**) GND map of small SF grains, (**j**) grain orientation of small SF grains, (**k**) SF value of small SF grains, and (**l**) PF of small SF grains.

**Figure 9:**
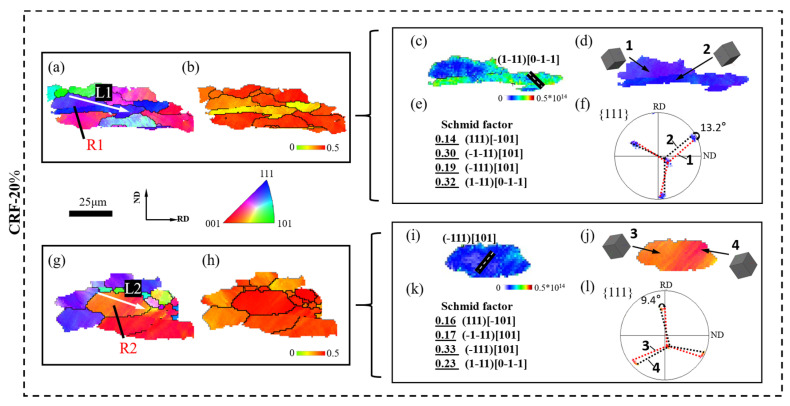
EBSD analysis of local area under CRF-20% conditions: (**a**) IPF map of large SF grains, (**b**) SF map of large SF grains, (**c**) GND map of large SF grains, (**d**) grain orientation of large SF grains, (**e**) SF value of large SF grains, and (**f**) PFs of large SF grains; (**g**) IPF map of small SF grains, (**h**) SF map of small SF grains, (**i**) GND map of small SF grains, (**j**) grain orientation of small SF grains, (**k**) SF value of small SF grains, and (**l**) PF of small SF grains.

**Figure 10:**
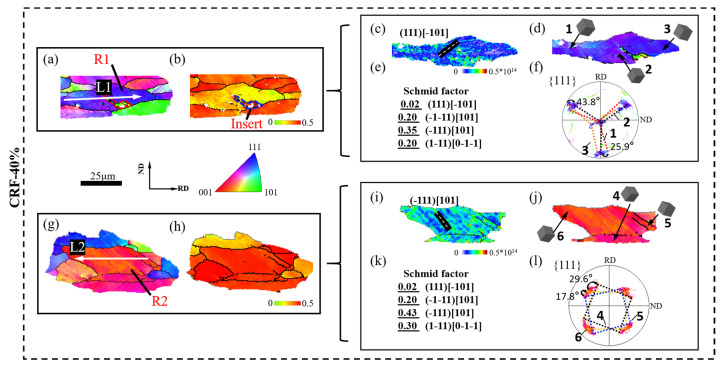
EBSD analysis of local area under CRF-40% conditions: (**a**) IPF map of large SF grains, (**b**) SF map of large SF grains, (**c**) GND map of large SF grains, (**d**) grain orientation of large SF grains, (**e**) SF value of large SF grains, and (**f**) PFs of large SF grains; (**g**) IPF map of small SF grains, (**h**) SF map of small SF grains, (**i**) GND map of small SF grains, (**j**) grain orientation of small SF grains, (**k**) SF value of small SF grains, and (**l**) PF of small SF grains.

**Figure 11:**
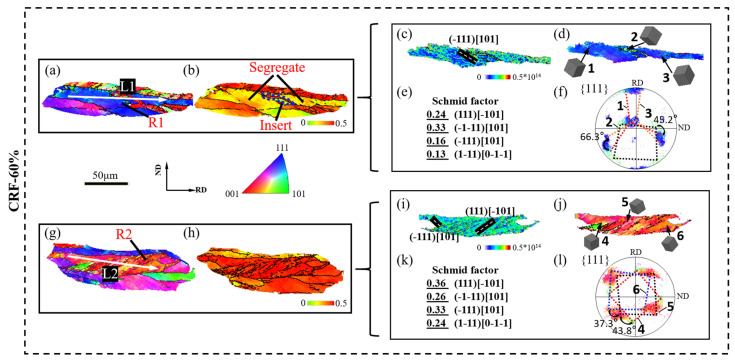
EBSD analysis of local area under CRF-60% conditions: (**a**) IPF map of large SF grains, (**b**) SF map of large SF grains, (**c**) GND map of large SF grains, (**d**) grain orientation of large SF grains, (**e**) SF value of large SF grains, and (**f**) PFs of large SF grains; (**g**) IPF map of small SF grains, (**h**) SF map of small SF grains, (**i**) GND map of small SF grains, (**j**) grain orientation of small SF grains, (**k**) SF value of small SF grains, and (**l**) PF of small SF grains.

**Figure 12:**
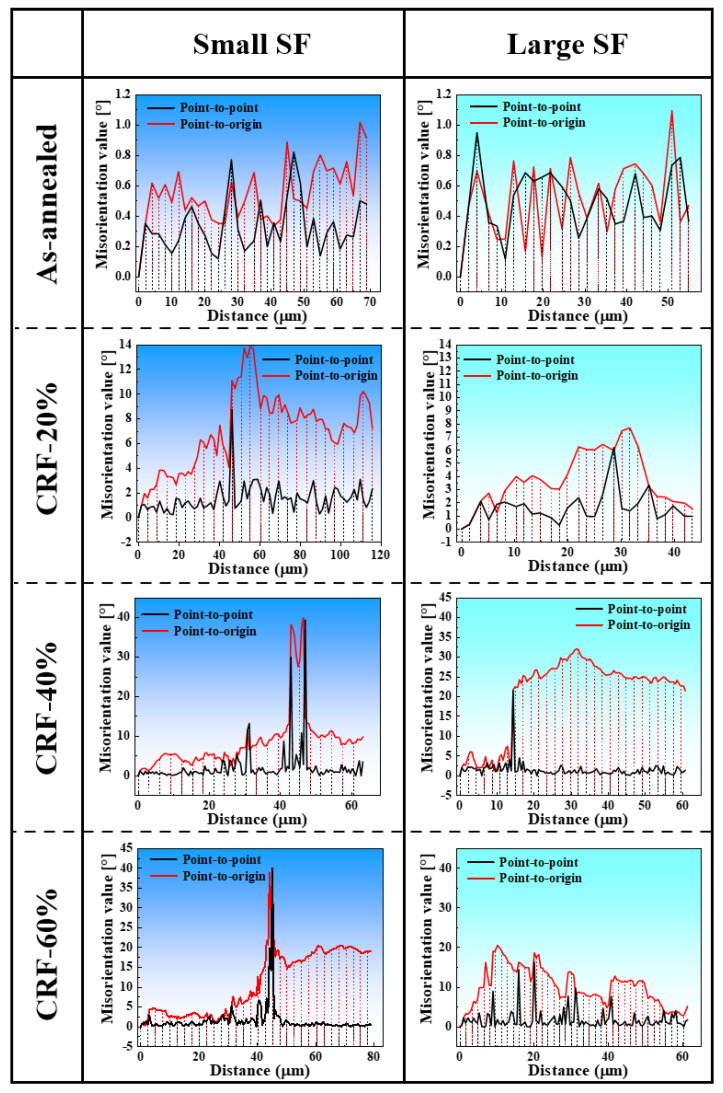
The misorientation values within large SF and small SF grains.

According to previous research [28,29,30], plastic deformation could promote slip behavior in aluminum matrix grains, which causes the migration of dislocation structures along slip traces and the formation of dislocation cell structures. With a large deformation amount, the misorientation across dislocation cell structure boundaries rapidly increases and grain boundaries are newly formed, leading to effective grain refinement. Therefore, the slip behaviors in small SF grains and large SF grains are further analyzed to discuss the grain refinement mechanism of 5A06 aluminum alloy sheets during the CRF process. The SF distribution, lattice orientation and slip system activation of small SF grains and large SF grains under different deformation conditions are analyzed, as shown in Figure 8, Figure 9, Figure 10 and Figure 11. R1 and R2 represent small SF grains and large SF grains, respectively, and the R1 and R2 at each deformation amount represent different grain refinement mechanisms. Moreover, the point-to-point misorientation and point-to-origin misorientation along selected lines are extracted, as shown in Figure 12. L1 and L2 represent the selected lines in R1 and R2, respectively.

Figure 8a–l shows the slip behavior under as-annealed conditions. Owing to the sufficient annealing effect, the dislocation density inside each grain is extremely low (Figure 8c,i). Moreover, their PFs (Figure 8f,l) exhibit a uniform lattice orientation, and no microbands can be found in grain orientation figures. Accordingly, L1 and L2 (Figure 12) both exhibit extremely low misorientations (<1°), which confirms the negligible orientation gradients under as-annealed conditions.

Figure 9a–l shows the slip behavior under CRF-20% conditions. Owing to initial slip deformation, parallel microbands appear in each grain, as shown in Figure 9a,g. However, compared to the single activated slip system ((−111) [101]) in R2, the GND figure of R1 exhibits two different activated slip systems ((1–11) [0–1–1] and (−1–11) [101]) in its different interior regions, which is mainly due to the higher CRSS of the slip system activation in R1 (hard orientation). The multi-slip behavior promotes the more severe dislocation multiplication in local regions of R1, and promotes comparatively more severe lattice rotation in R1, as confirmed by the lattice rotation values in PFs (Figure 9f,l). During more severe lattice rotation, the misorientation angle across the microband boundaries in R1 gradually increases, resulting in initial grain boundary formation. L1 (Figure 12) exhibits a maximum point-to-point misorientation of 8.6°, and the point-to-origin misorientation increases overall after crossing a newly formed grain boundary, which indicates the influence of non-uniform slip behavior on grain boundary formation. In contrast, the uniform single slip deformation in R2 causes very weak dislocation multiplication and uniform microband distribution. Accordingly, both point-to-point and point-to-origin misorientations along L2 (Figure 12) are uniformly low, which indicates that the uniform single slip deformation has little influence on grain boundary formation in large SF grains under CRF-20% conditions.

Figure 10a–l shows the slip behavior under CRF-40% conditions. As can be seen, both R1 and R2 experience more severe slip deformation. However, the slip behaviors of R1 and R2 are different. R1 exhibits multi-slip system activation in different interior regions, and the maximum lattice rotation angle is 43.8°. During this non-uniform slip deformation, different interior regions of R1 cannot maintain overall coordinated movement, leading to non-uniformly distributed dislocations and high strain localization in R1. New grain boundaries tend to pre-form along single microbands with a comparatively higher dislocation density. With the combination of a dense dislocation and a hard orientation, R1 experiences initial separation along the pre-formed grain boundary, and its adjacent large SF grain directly inserts in the interior vacancy of R1. R2 still exhibits single slip system activation, and the lattice rotation angle clearly increases compared to CRF-20% conditions. However, owing to the soft orientation of R2, dislocations can multiply and migrate smoothly along different microbands. Consequently, dislocation aggregation only occurs along very few microband boundaries, which causes initial HAGB formation along single microband boundaries and mass LAGB formation along most microband boundaries. On the one hand, the misorientation angles along L1 and L2 confirm new grain boundary formation. On the other hand, the point-to-origin misorientation angles indicate that more non-uniform slip deformation occurs in both R1 and R2 under these deformation conditions.

Figure 11a–l shows the slip behavior under CRF-60% conditions. As can be seen, both R1 and R2 experience strong elongation, and slip deformation is even more severe in these elongated grains. The maximum lattice rotation angle in R1 reaches 66.3°, and most of the activated slip systems in R1 are parallel to the (−1–11) [101] slip direction. The dislocation density is uniformly high in R1, which contributes to strain localization in several local regions. Owing to the hard orientation and uniformly dense dislocation, the coordinate movement between different interior regions is completely restrained, and R1 exhibits complete separation along its pre-formed grain boundary. Meanwhile, several microbands of adjacent large SF grains fully insert into the interior vacancies of R1. R2 exhibits multi-slip system activation, more than those with smaller deformation amounts (Figure 11i). Accordingly, the lattice rotation in R2 becomes stronger, and the maximum lattice rotation angle reaches 43.8°. Alongside severe lattice rotation, rapid dislocation, multiplication and migration occur, resulting in a uniform misorientation increasing along the microbands and grain boundary formation inside R2. The point-to-point misorientation angle along L2 confirms the multiple grain boundary formation and intermediate point-to-origin misorientation angles (<20°) along the grain interior part of L1 and L2 confirm the uniform and strong slip deformation in the final deformation stage.

**Figure 13:**
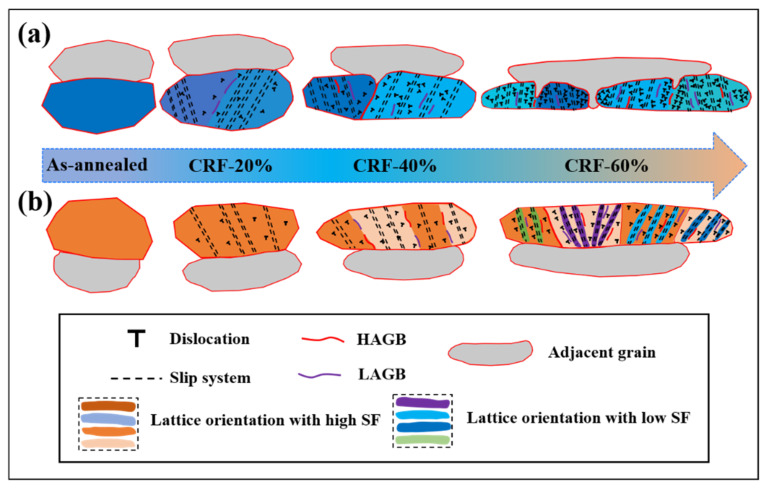
Schematic diagram of the grain refinement mechanism of 5A06 aluminum alloy sheets during the CRF process: (**a**) grains with a low SF and (**b**) grains with a high SF.

The present study shows that effective grain refinement can be achieved for 5A06 aluminum alloy sheets by CRF deformation, and the lattice orientation of matrix grains plays a vital role in the grain refinement mechanism. In summary, the grain refinement mechanism can be divided into two modes: (1) Small SF grain refinement (Figure 13a). Under as-annealed conditions, the small SF grains possess an extremely low dislocation density. Owing to the hard lattice orientation, slip system activation requires a larger CRSS in these small SF grains. Therefore, during the initial CRF process, multiple slip systems are activated in different interior regions, and parallel microbands form in each region with different slip systems. Meanwhile, dislocation rapidly multiplies and migrates along the boundaries of these regions, leading to the initial LAGB formation. With the increasing CRF deformation amount, more severe lattice rotation and dislocation segregation occur along single microbands, which leads to the pre-formation of HAGBs. Moreover, strain localization restrains coordinate slip deformation, leading to the initial separation of small SF grains along pre-formed HAGBs. The adjacent grains directly insert into the interior vacancies of small SF grains. In the final CRF deformation stage, the influence of a combination of a more severe lattice rotation and dislocation segregation further exacerbates the strain localization along the pre-formed HAGBs, causing complete separation of small SF grains along the pre-formed HAGBs. Consequently, the interior vacancies of these small SF grains are completely filled by adjacent grains. Meanwhile, owing to the rapid dislocation, multiplication and migration, further grain refinement occurs inside refined small SF grains. (2) Large SF grain refinement (Figure 13b). Large SF grains under as-annealed conditions possess an extremely low dislocation density, which is similar to small SF grains. Owing to the soft orientation, the slip system can be easily activated and dislocation can migrate smoothly throughout these large SF grains. Therefore, during the initial CRF process, a small lattice rotation is required to promote slip deformation and uniform slip behavior occurs in different interior regions, causing single slip system activation and uniform microband formation. Moreover, low-density dislocations are uniformly distributed inside these larger SF grains. With the increasing CRF deformation amount, more severe lattice rotation occurs to promote slip deformation, leading to a slight increase in misorientation across microband boundaries. However, owing to the smooth dislocation migration, only a small number of dislocations segregate along some microbands. Therefore, LAGB formation is dominant under these deformation conditions. In the final CRF deformation stage, owing to the gradual increase in dislocation density, even more severe lattice rotation is required to maintain slip deformation, which accelerates the lattice rotation towards a hard orientation. Under the influence of a combination of hard-orientation transformations and dislocation segregation, multi-slip system activation occurs in different interior regions. This non-uniform slip behavior leads to rapid misorientation, increasing across microband boundaries and thus causing mass HAGB formation. Consequently, many fine grains are directly formed inside original matrix grains, and these refined grains are bonded as single cell blocks.

## 4. Conclusions

In summary, the grain structure evolution of 5A06 aluminum alloy during the CRF process is investigated. During the CRF process, the average GND value increases from 0.17 × 10^14^ to 4.63 × 10^14^, and the GND standard deviation values of small SF grains are consistently higher than those of large SF grains, which indicates non-uniform deformation in small SF grains. Effective grain refinement is achieved, and the average grain size decreases from 25.1 µm to 11.8 µm. Grains with different lattice orientations exhibit different slip system activation modes and different dislocation movements, which is primarily responsible for the different grain refinement mechanisms. The grain refinement mechanism can be divided into two modes: (1) The grains with a small SF get multi-slip systems activated, and dense dislocations are segregated along the boundaries of interior regions with different slip systems, which results in a rapidly increasing strain localization along these boundaries. Since the strain localization restrains the coordinate slip deformation between different interior regions, the grains are directly separated into several finer grains. (2) The grains with a large SF primarily are activated by a single slip system, and the dislocation migrates smoothly along most microband boundaries. Then, a more severe lattice rotation causes a transformation to a hard orientation and multi-slip system activation, which contributes to the increase in rapid misorientation across the microband boundaries and thus promotes significant SF grain refinement.

## Figures and Tables

**Table 1:** Measured chemical composition of 5A06 aluminum (wt%).

Mg	Mn	Fe	Si	Ti	Cr	Al
5.943	0.621	0.175	0.136	0.038	0.034	Bal.

## Data Availability

This study does not use other people’s research data, and the data in this paper are all obtained through experiments.
